# A miRNA signature assessing ovarian cancer prognosis

**DOI:** 10.18632/oncoscience.329

**Published:** 2016-12-02

**Authors:** Marina Bagnoli, Sandro Pignata, Delia Mezzanzanica

**Affiliations:** Molecular Therapies Unit, Dept of Experimental Oncology and Molecular Medicine, Fondazione IRCCS Istituto Nazionale dei Tumori Milan, Milan, Italy

**Keywords:** ovarian cancer, microRNA, MITO translational group, early relapse, miR-506 family

Epithelial ovarian cancer (EOC) is the commonest cause of gynecological cancer-related death; it is usually diagnosed at late stages when it is widely spread into the peritoneal cavity. Standard care remains surgery followed by platinum-based chemotherapy and despite a good response rate to front-line treatment, most of the patients eventually develop an incurable state of platinum-resistant disease [1]. This underlines the need of identifying prognostic biomarkers to improve our understanding of the molecular bases of chemoresistance and disease progression with the final aim of selecting EOC patients with very unfavorable prognosis to improve the design of tailored therapies.

In the era of next generation sequencing, to provide information on tumor progression and relapse, we decided to rely on the importance of expression studies, in particular on the non coding area of the genome. Indeed, we have focused our attention to microRNAs (miRNAs), short (21-25 nucleotides) non coding RNA that regulate the expression of about 60% of genes and whose hairpin structure makes them particularly resistant to degradation and stable in cells and in body fluids and therefore attractive candidates as cancer biomarkers [2].

By using 3 retrospective cohorts including 894 EOC cases, which is the largest collection so far analyzed for miRNA expression, we derived a robust 35 miRNAbased predictor of EOC risk of relapse (MiROvaR) [3]. Through the collaboration with the Multicenter Italian Trial in Ovarian (MITO) group, we developed MiROvaR by using as training set (OC179), samples derived from the randomized phase III MITO2 clinical trial (see 3). On this cohort MiROvaR was able to stratify patients for their risk of relapse/progression with a difference in the median progression free survival (PFS) of 20 months between high and low risk groups [3]. Our model, which is available to the scientific community, has been validated in two independent cohorts, one collected in our Institutions (OC263) and the other derived from The Cancer Genome Atlas (TCGA) [4], the only so far available public EOC collection with fully annotated clinical data. The lack of such information in other miRNA data sets, none of which derived from clinical trials, limited our possibility to further validate MiROvaR. We are aware that the patients' populations included in training set and in OC263 validation set are more heterogeneous as compared to the TCGA data set, which includes only high grade serous ovarian cancer. However, at variance from TCGA data set, OC179, being derived from a clinical trial, is extremely well clinically annotated. On the other end, the reproducibility of TCGA Ovarian cancer miRNA profiles has been recently questioned [5]. This may account for the fact that, although significant, the difference in the median PFS between high and low risk groups was of 4 months only in TGCA collection [3]. However, maintenance of MiROvaR independent prognostic impact in multivariable analysis including the strongest prognostic clinical variables for EOC in terms of PFS prediction (i.e. FIGO stage and residual disease), support its usefulness in prognostic classification of EOC patients, regardless the biological/molecular differences among histotypes.

Our effort to globally analyze 894 EOC samples for miRNA expression profile has implicated the need for an accurate data pre-processing to allow the best comparison among the different platforms and chip arrays used to obtained the analyzed profiles. After data filtering and miRNAs re-annotation, we obtained a list of 385 unique miRNAs shared among the platforms from which the 35 miRNA-based predictor of EOC risk of relapse was developed [3]. By relying on the 385 miRNA shared by all the used platforms we may have lost other important miRNAs but we believe that our work is one of the few attempts in integrating the existing data trying to overcome one of the limitation related to miRNA analysis: the use of different platforms and different annotated lists. Although only a shared list of 385 miRNA could be tested, we expect that during MiROvaR further validations, in the case other pivotal miRNAs will be indentified, they can be tested and integrated into the signature with the purpose to refine the number of miRNAs entering into a possible clinical-grade tool. The miRNA signature will be further corroborated by a concurrent gene expression analysis of our case materials to shed light in to the signaling pathways involved in the biological processes regulated by MiROvaR and apparently associated to epithelial mesenchymal transition (EMT). Indeed among the major contributors to MiROvaR performance there are miRNA belonging to the miR-506 family that we have previously identified as down-modulated in early relapsing EOC patients [6] and involved in regulation of keys EMT nodes and response to therapy [7,8].

Beyond standard clinical parameters (stage, histotype and grade), there are few factors able to support clinicians in identifying those EOC patients who would indeed benefit from alternative therapeutic modalities.

**Figure 1:**
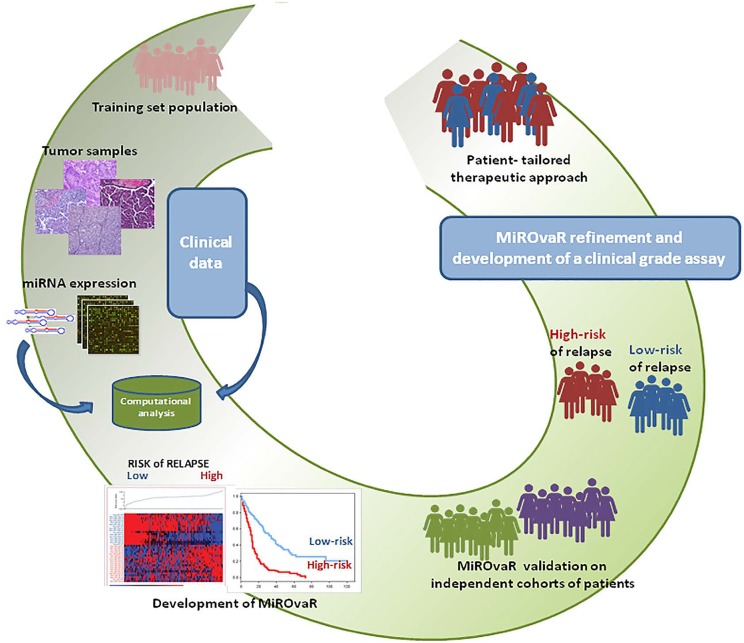
Roadmap of MiROvaR development and application.

This study, showing that the prognostic role of MiROvaR can be shared across different histological subtypes, represent an important step forward in highlighting the role of miRNAs as biomarkers in EOC and warrant a further prospective validation for MiROvaR entering into clinical practice. Concerning its applicability, the subgroup of advanced stage EOC patients with a very unfavorable prognosis identified by MiROvaR might be candidated to more aggressive strategies (addition of bevacizumab and/or maintenance treatment in first line therapy). On the other hand, if applied to early stages EOC patients MiROvaR can help in identifying those patients who could really benefit from chemotherapy, sparing low risk patients from over-treatment.
